# Development of YOLOv5-Based Real-Time Smart Monitoring System for Increasing Lab Safety Awareness in Educational Institutions

**DOI:** 10.3390/s22228820

**Published:** 2022-11-15

**Authors:** Luqman Ali, Fady Alnajjar, Medha Mohan Ambali Parambil, Mohammad Issam Younes, Ziad Ismail Abdelhalim, Hamad Aljassmi

**Affiliations:** 1 Department of Computer Science and Software Engineering, College of Information Technology, United Arab Emirates University (UAEU), Al Ain 15551, United Arab Emirates; 2 Emirates Center for Mobility Research, United Arab Emirates University (UAEU), Al Ain 15551, United Arab Emirates; 3 AI and Robotics Lab (Air-Lab), United Arab Emirates University (UAEU), Al Ain 15551, United Arab Emirates; 4 Department of Civil Engineering, College of Engineering, United Arab Emirates University (UAEU), Al Ain 15551, United Arab Emirates

**Keywords:** IoT, smart academic laboratories, safety, deep learning, object detection, YOLOv5, PPE compliance

## Abstract

The term “smart lab” refers to a system that provides a novel and flexible approach to automating and connecting current laboratory processes. In education, laboratory safety is an essential component of undergraduate laboratory classes. The institution provides formal training for the students working in the labs that involve potential exposure to a wide range of hazards, including chemical, biological, and physical agents. During the laboratory safety lessons, the instructor explains the lab safety protocols and the use of personal protective equipment (PPE) to prevent unwanted accidents. However, it is not always guaranteed that students follow safety procedures throughout all lab sessions. Currently, the lab supervisors monitor the use of PPE, which is time consuming, laborious, and impossible to see each student. Consequently, students may unintentionally commit unrecognizable unsafe acts, which can lead to unwanted situations. Therefore, the aim of the research article was to propose a real-time smart vision-based lab-safety monitoring system to verify the PPE compliance of students, i.e., whether the student is wearing a mask, gloves, lab coat, and goggles, from image/video in real time. The YOLOv5 (YOLOv5l, YOLOv5m, YOLOv5n, YOLOv5s, and YOLOv5x) and YOLOv7 models were trained using a self-created novel dataset named SLS (Students Lab Safety). The dataset comprises four classes, namely, gloves, helmets, masks, and goggles, and 481 images, having a resolution of 835 × 1000, acquired from various research laboratories of the United Arab Emirates University. The performance of the different YOLOv5 and YOLOv7 versions is compared based on instances’ size using evaluation metrics such as precision, F1 score, recall, and mAP (mean average precision). The experimental results demonstrated that all the models showed promising performance in detecting PPE in educational labs. The YOLOv5n approach achieved the highest mAP of 77.40% for small and large instances, followed by the YOLOv5m model having a mAP of 75.30%. A report detailing each student’s PPE compliance in the lab can be prepared based on data collected in real time and stored in the proposed system. Overall, the proposed approach can be utilized to make laboratories smarter by enhancing the efficacy of safety in research settings; this, in turn, will aid the students in establishing a health and safety culture among students.

## 1. Introduction

Regular classroom instructions and academic research are facilitated by labs at institutions, which are responsible for training future scientists and uncovering the mysteries of nature [1]. Several recent incidents in university laboratories have prompted an increased focus on laboratory safety [2,3,4,5]. Universities and science departments have taken various initiatives, such as performing multiple training sessions and safety plans and providing online information to ensure the safety protocols in the lab environment. Maintaining active involvement in safety training by principal investigators (PIs) or lab supervisors is critical to fostering good attitudes toward academic laboratory safety. Traditionally, the lab instructors arrange training sessions for newcomers to maintain a safe learning and working environment for their students. In the training sessions, the lab instructors guide the students about the lab safety protocol and the use of PPE. The personal protective equipment (PPE), which includes safety eye wear, lab coat, gloves, and mask, provides a direct protective layer to the students. The use of PPE in the laboratories indicates the extent to which students are following the safety policies of the institution. Previous studies showed that PPE compliance by researchers in academia was positively influenced when their safety behavior was monitored [6]. 

Traditionally, the PPE is manually monitored by lab supervisors; the monitoring is expensive, time consuming, and resource intensive. The instructors cannot keep a vigilant eye on every student and track their movements during the lab sessions to ensure PPE compliance. Additionally, it is challenging to fulfill the requirements of modern educational safety management by solely relying on manual monitoring approaches. To bridge these problems, automatic vision-based deep learning monitoring and detection techniques provide the solution. These approaches have shown promising performance in tackling the challenge of accurate safety monitoring and hazard detection problems in various applications [7,8,9,10,11,12,13,14]. Most studies focus on detecting and monitoring PPE compliance for workers’ safety in the construction industry [15,16,17]. Several different industries, such as construction, mining, and energy, have started to make investments in order to improve the safety of their workers by incorporating new technology, sometimes known as “smart technologies”, into the workplace environment. These technologies are in charge of keeping an eye on workers and ensuring their safety in the workplace. Wu et al. [18] used the Single Shot MultiBox Detector (SSD) [19] for the detection of construction workers’ helmets and their associated colors. Fang et al. [20] proposed an end-to-end Faster RCNN [21]-based approach for the non-hard-hat-use (NHU) detection in raw videos. Similarly, Saudi et al. [22] used the Faster RCNN method for detecting multiple PPEs such as helmets and vests for the safety of the worker in construction sites. 

**Figure 1:**
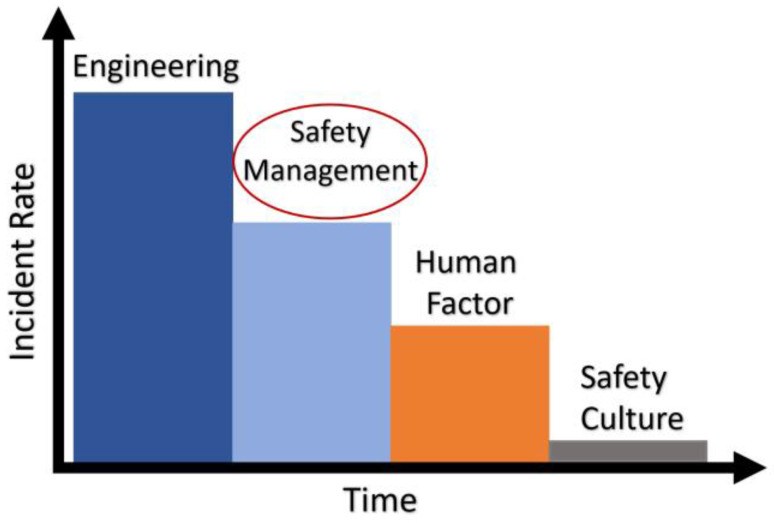
Role of safety culture in reducing incidents’ rate.

The YOLO architecture is becoming increasingly popular because of its speed and precision in the recognition of objects from images. Nath et al. [23] proposed a real-time You-Only-Look-Once (YOLO) architecture for the verification of PPE compliance of workers, i.e., whether or not a worker was wearing a hard hat, vest, or both. Moreover, human identity recognition and helmet detection was performed using YOLOv3 architecture in [24]. Wang et al. [25] compared the performance of various architectures of the YOLO family (YOLOv3 [16], YOLOv4 [26], and YOLOv5 [27]) on a custom dataset, named as the CHV dataset, and found that YOLOv5x had a superior performance as compared to other models. Among all real-time object detectors with 30 FPS or higher on GPU V100, OLOv7 had the best accuracy (56.8% AP) and was the fastest (up to 160 FPS) [28]. To the authors’ knowledge, minimal efforts were made to use these object detection models for the detection of PPE in educational laboratories. Therefore, this paper proposes a YOLOv5-based real-time PPE compliance detection and monitoring system for academic laboratories using a custom dataset. The main aim of the system is to create a reliable and real-time automated smart safety detection system by having early warning systems, which will create a safety culture in the institutional lab, enhance the lab safety awareness, and eliminate the occurrence of various unwanted incidents, as depicted in Figure 1. The data of the student regarding the student will be sent to the system and a safety report for the student will be generated. The system can also be set up at the entrance door to the laboratory, allowing only properly geared-up personnel access. Students are not allowed inside the lab and are informed of their error if they are found to be lacking proper PPE. The main aims of the proposed study are:

(1) The creation of a novel, labeled PPE dataset named SLS (Student Lab Safety) containing four different classes, including mask, lab coat, safety glass, and gloves. The dataset contains 481 images and the corresponding annotations of these four classes.
(2) The performance evaluation of various versions of the YOLOv5 [27] (YOLOv5l, YOLOv5m, YOLOv5n, YOLOv5s, and YOLOv5x) and YOLOv7 (YOLOv7 and YOLOv7X) on the proposed dataset for the detection and monitoring of students’ PPE in academic laboratories.
(3) The performance evaluation of the YOLOv5 and YOLOv7 model variant based on instance size of the object, i.e., large instances (lab coat and gloves) and small instances (masks and goggles).

## 2. System Overview 

**Figure 2:**
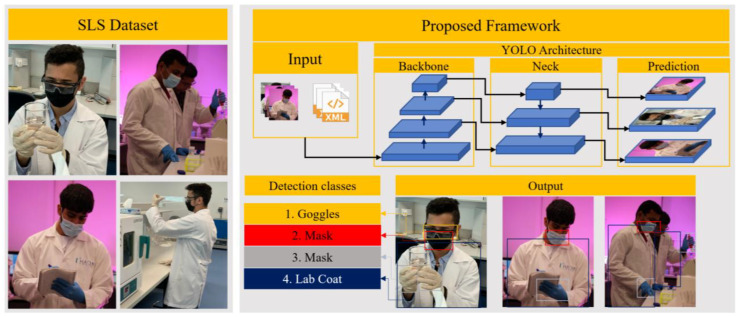
Overview of the proposed system (correct the arrows).

The overview of the proposed system is depicted in Figure 2: (1) SLS dataset Preparation, (2) training of the proposed YOLOv5 and YOLOv7 frameworks, and (3) testing of the proposed system in real-time environments. The system takes images/videos as input and analyzes them using the trained versions of the YOLOv5 and YOLOv7 models. The output image/video from the system contains different detected classes, i.e., gloves, lab coat, goggles, and mask. Each section of the proposed system is explained in detail below. 

### 2.1. Student Laboratory Safety (SLS) Dataset 

**Figure 3:**
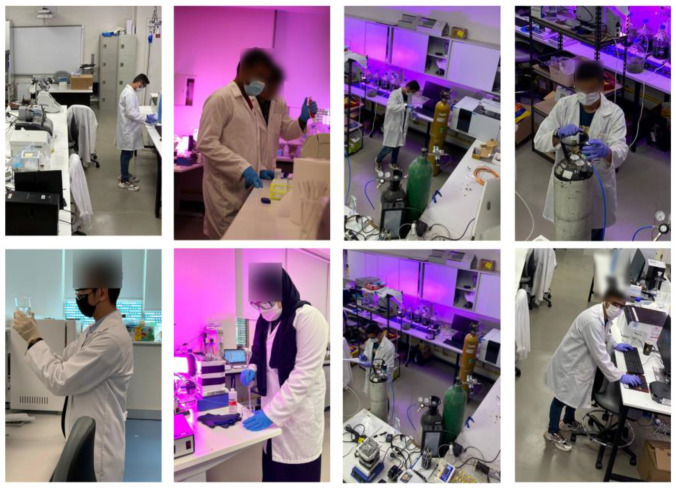
Sample images of Student Laboratory Safety (SLS) dataset.

In the proposed study, a novel dataset named SLS was created for PPE detection as there is currently no publicly accessible dataset for the detection of PPE in educational labs. The images were acquired from students working in research laboratories of the United Arab Emirates University using a handheld Canon EOS 650D (40 mm) and surveillance (CCTV 2.0 Dome Camera PoE Onvif conformant) camera installed in the lab. The images were taken from various distances and viewpoints of students working in lab surroundings including the top camera installed in the lab. In order to protect the identity of the students working in the laboratory, their faces were obscured. The dataset contained 481 images having a resolution of 1600 × 1200 pixels collected from the handheld and surveillance cameras installed in the lab. The activities of the students were divided into two categories: those who followed PPE compliance were marked safe and those who did not were marked unsafe. The number of images in both categories was kept equal. After data acquisition, the images were manually labeled using the graphical images annotation tool LabelImg [29]. The labeled dataset contained 1485 instances of masks, gloves, goggles, and lab coat, each with a class label and bounding box. The number of instances for class gloves, lab coat, goggles, and masks were 421, 421, 322, and 321, respectively. The number of small-scale instances was greater than large-scale instances, which made the job of PPE detection more challenging. Object detection models require a large number of samples to train; therefore, data augmentation was applied on the original dataset to increase the size of the dataset using Roboflow [30]. After data augmentation, a total of 1164 images, which were 931, 116, and 116 images, were used for training, validation, and testing, respectively. After data augmentation, the images were resized to 416 × 416 pixels. The sample images of the SLS dataset are shown in Figure 3. The acquired dataset was then given to various variants of the YOLOv5 and YOLOv7 models for training the PPE detection system. 

### 2.2. YOLOv5 Model 

**Figure 4:**
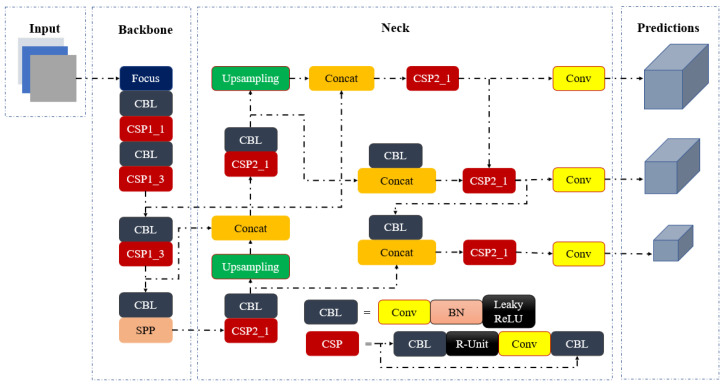
Network structure of YOLOv5.

The R-CNN [31], Faster RCNN [21], and YOLO [32] series are currently the most popular object detection algorithms in research. The YOLO series is superior to the earlier models in terms of its increased speed and its capacity to detect small objects. In this work, both training and testing were performed by using various versions of the YOLOv5 models. YOLOv5 [27], released in 2020, provides a variety of object identification architectures that have already been trained using the MS COCO dataset. There are five distinct versions of YOLOv5, ranging from the tiny YOLOv5 nano version, designed for use on mobile and embedded devices, to the massive YOLOv5x large version. The YOLOv5 architecture is composed of various components, including the backbone, neck, and head, as shown in Figure 4.

**Figure 5:**
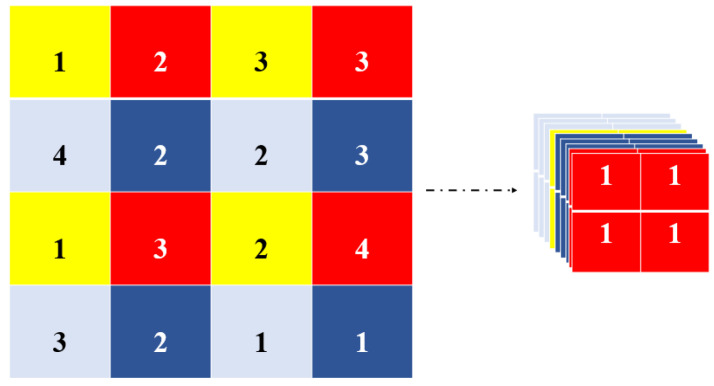
The processing flow of focus module.

The backbone consists of the focus structure [33] and Cross Stage Partial Networks (CSP) [34]. The focus structure downsamples the input data dimension while preserving the original information, as shown in Figure 5. 

**Figure 6:**
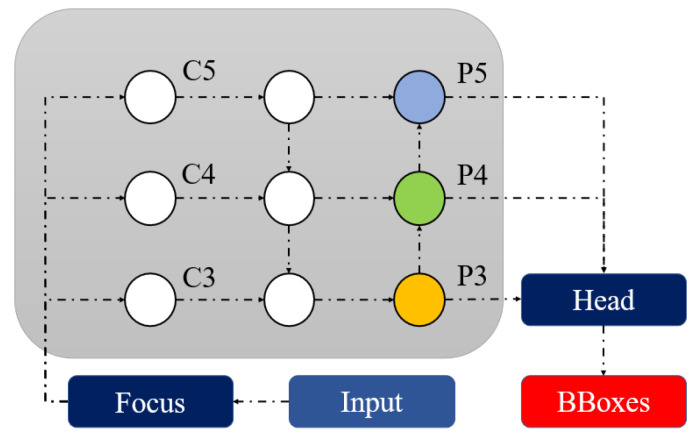
Representation of feature fusion of YOLOv5 model.

The CSP Network extracts useful information, which improves the learning ability and reduces the memory cost of the model. The neck part combines the acquired features and forwards them to the prediction layer by using Feature Pyramid Networks (FPN) and the Path Aggregation Network (PAN). The FPN upsamples the high-level feature information through top-to-bottom communication and fusion for prediction. The underlying pyramid, PAN, conveys significant positional characteristics from a bottom-to-top manner, which helps in the differentiation of the same objects with different sizes and scales. The feature pyramids help the model to perform efficiently on new data. Figure 6 depicts how the feature extraction network upsamples its output feature map (F1, F2, and F3) by generating numerous new feature maps (P1, P2, and P3) for recognizing targets of varying scales. The output layer, the head, generates the final output vectors by applying anchor boxes on features and generates the final output vector, which includes class probabilities, object scores, and bounding boxes. The addition of the focus and CSP layers is the most notable improvement in YOLOv5. The focus layer reduces layers, parameters, FLOPS, and CUDA memory to increase forward and backward speeds. The CSP layer used as the backbone layer aims to extract detailed information and perform more comprehensive tasks. The meshing concepts of the original YOLO algorithm have been carried over into YOLO v5. 

The network takes an RGB image as an input and produces a three-scale (small, medium, and large) output. The process of bounding box regression of YOLOv5 can be explained in detail by Equation (1) [35].

(1) $$b_{x}=2\sigma \left(s_{x}\right)-\frac{1}{2}+r_{x}\;b_{y}=2\sigma \left(s_{y}\right)-\frac{1}{2}+r_{y}\;b_{h}=p_{h}{\left(2\sigma \left(s_{h}\right)\right)}^{2}\;\;b_{w}=p_{w}{\left(2\sigma \left(s_{w}\right)\right)}^{2}$$

**Figure 7:**
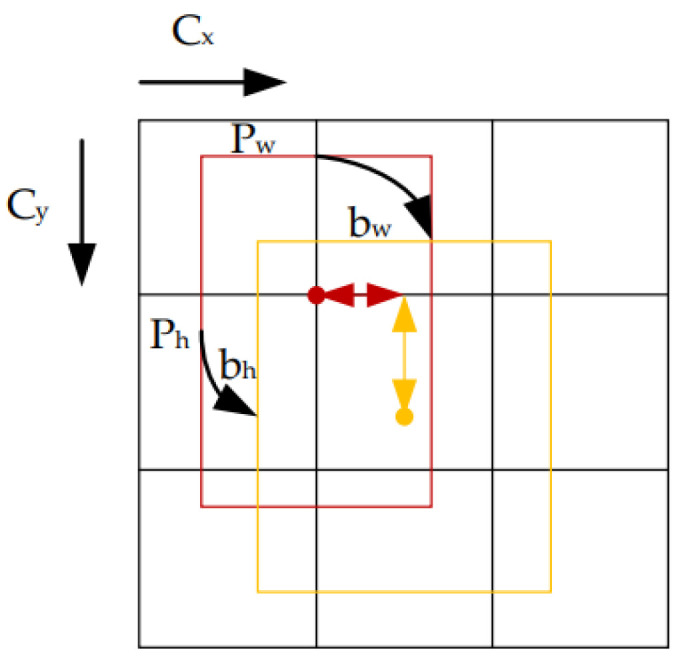
Decoding of prediction bounding box in YOLOv5, acquired from [35].

In the above equation, the coordinate value of the upper left corner of the feature map should be set to (0, 0). The values $r_{x}$ and $r_{y}$ represent the distance between the center of the label bounding box and the upper left corner of the grid, respectively. The $b_{x}$ and $b_{y}$ are the center point coordinates while $b_{h}$ and $b_{w}$ represent the width and height of the label bounding box, as shown in Figure 7. The prior bounding box width and height are represented by $p_{h}$ and $p_{w}.$ The $s_{x}$, $s_{y}$, $s_{h},$ and $s_{w}$ are parameters related to the bounding box. 

### 2.3. YOLOv7 Model 

The most recent YOLO object detection model, YOLOv7 [28], was developed by Alexey Bochkovskiy. The architecture surpasses all the previous versions in terms of detection accuracy and speed. The authors’ primary contributions to the YOLOv7 model that allowed it to reach this pinnacle were: (1) their ultimate aggregation layer, E-ELAN, is an enhanced form of the efficient layer aggregation (ELAN) computational block; (2) model depth and breadth can be scaled in parallel by concatenating layers, an innovative approach to model scaling; and (3) the introduction of an auxiliary head network to enhance the training process and model re-parameterization technique to make the model more resilient and generalize well on fresh data. 

## 3. Experimental Results

### 3.1. Environmental Setup

The proposed system was trained using NVIDIA DGX-1, “The Fastest Deep Learning System” for AI Research based at the AI and Robotics Lab of United Arab Emirates University. The system consists of dual 20-core Intel^®^ XEON^®^ E5-2698 v4 2.2 GHz CPUs and 40,960 NVIDIA CUDA cores. The system has 8× Tesla V100 GPUs and a total of 256 GB GPU Memory. PyTorch library, Windows 10, and Python3.8 were used to train the YOLOv5 and YOLOv7 models and accomplish the predictions. The performance of the model was evaluated by using various evaluation metrics, each of which is explored in further depth in the next section.

### 3.2. Evaluation Metrics 

In the proposed study, various evaluation metrics such as precision, recall, average precision (*AP*), mean average precision (*mAP*), and intersection over union (*IoU*) were used to compare the acquired experimental results. The term “intersection over union,” which is given in Equation (2), describes the degree to which two bounding boxes, i.e., predicted $\left(PR\right)$ and ground-truth $\left(GT\right)$, overlap one another. The higher the IoU, the larger the area of overlap should be.

(2) $$IOU=\frac{Area\;of\;intersection}{Area\;of\;union}=\frac{Area\;\left(GT\cap PR\right)}{Area\;\left(GT\cup PR\right)}$$

A recall is the true positive rate, which is also known as sensitivity and is a metric that determines how likely it is that ground-truth objects will be successfully recognized. A high recall is achieved by a model when it does not produce any false negatives, which means that there are no bounding boxes that are not detected but should be detected. The mathematical representation for the recall is given in Equation (3) below.

(3) $$R=\frac{TP}{TP+FN}=\;\;\;\;\frac{TP}{Total\;Ground\;Truths}$$

In the above equation, TP and TN represent the true positive and false negative, respectively. Precision, also known as the positive predictive value, defined in Equation (4), is the proportion of predicted positives that are correct. The precise model identifies only relevant objects and produces no false positives (*FP*).

(4) $$P=\frac{TP}{TP+FP}\;=\;\;\;\frac{TP}{Total\;Predictions}$$

The harmonic mean of the precision and recall scores is the *F*−1 score as defined in Equation (5).

(5) $$F-1=2*\frac{P*R}{P+R}$$

AP is the area under the precision–recall curve while mAP is the average of all AP values over different classes/categories, as shown in Equation (6).

(6) $$mAP=\frac{1}{n}\sum_{i=1}^{n}AP_{i}$$

where n is the number of classes.

### 3.3. Analysis of Experimental Results and Discussion 

**Figure 8:**
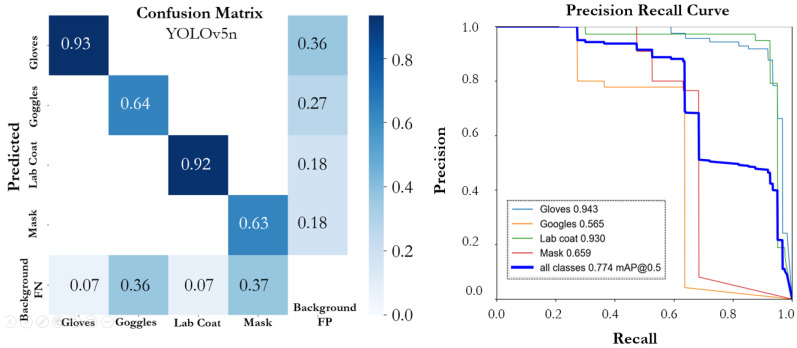
Confusion matrix and PR curve of the YOLOv5n model.

**Figure 9:**
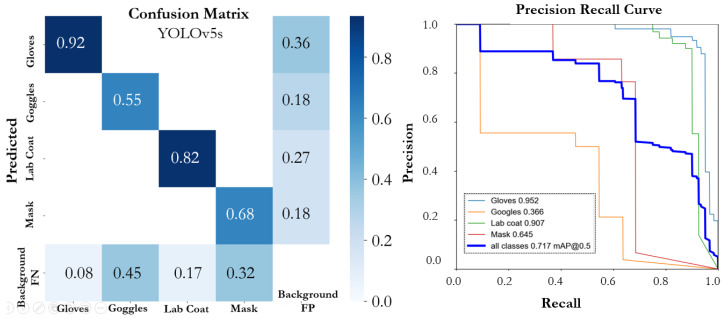
Confusion matrix and PR curve of the YOLOv5s model.

**Figure 10:**
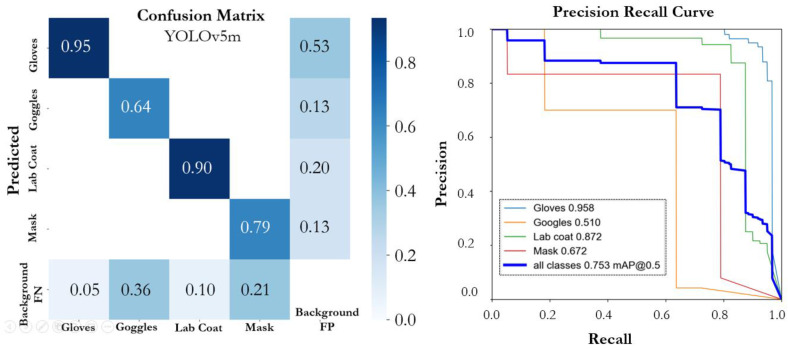
Confusion matrix and PR curve of the YOLOv5m model.

**Figure 11:**
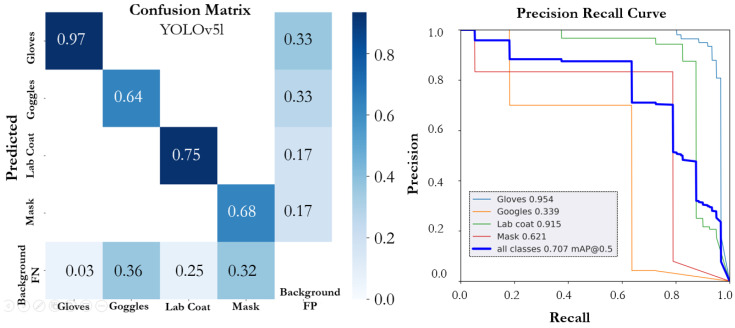
Confusion matrix and PR curve of the YOLOv5l model.

**Figure 12:**
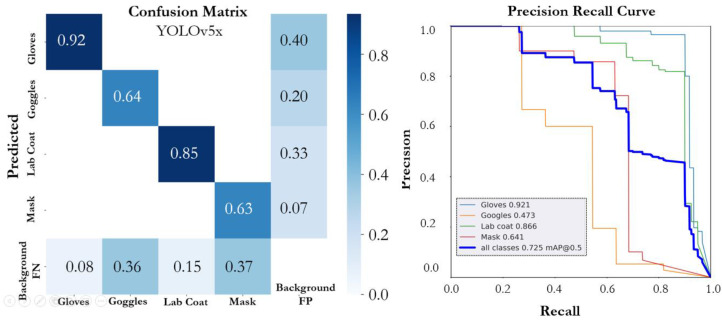
Confusion matrix and PR curve of the YOLOv5x model.

The performance of various YOLOv5 and YOLOv7 versions on the proposed SLS dataset is summarized in Table 1. The number of epochs that were considered necessary for training all of the models was 300; however, an early stopping condition was utilized to prevent model overfitting. All the models that were trained on the SLS dataset showed promising performance. The YOLOv5n model achieved an mAP@0.5 of 0.774 with a precision of 0.795 and a recall of 0.787. The individual class score of the YOLOv5 models and the performance of each model based on instance size is summarized in Table 2. The model (YOLOv5n) achieved the highest mAP@0.5, precision, and recall, of 0.943, 0.918, and 0.918, for the large-scale instance (lab coat) followed by the gloves, respectively. The YOLOv5n model size had the lowest size, of 3.9 MB, and had a faster inference time than the other compared models. The PR curve and confusion matrix of the model are shown in Figure 8. The YOLO5s model achieved an mAP@0.5, precision, and a recall of 0.717, 0.798, and 0.7022. In the individual class performance, the large-scale instances (lab coat and glasses) outperformed the other small-scale instances and achieved an individual mAP@0.5 of 0.907 and 0.952, respectively. Figure 9 depicts the confusion matrix and precision–recall curve of the YOLO5s model. The YOLOv5m model outperformed the YOLOv5s model in terms of mAP@0.5 and precision; however, the performance was lower than the YOLOv5n model. The model (YOLOv5m) achieved mAP@0.5, precision, and recall of 0.753, 0.776, and 0.837, respectively. The confusion matrix and PR curve of the model is shown in Figure 10. The YOLOv5l and YOLOv5x models achieved an mAP@0.5 of 0.707 and 0.725, respectively. The confusion matrices and PR curves of both models are shown in Figure 11 and Figure 12. It can be seen from the results that increasing the number of parameters had a significant effect on the detection performance of the safety system. The performance of the YOLOv5 model degraded moving from the YOLOv5n model, having a lower number of parameters, to the YOLOv5x model, which had a large number of parameters. An increasing number of parameters also affected the detection speed of the model. In the proposed work, the YOLOv5s and YOLOv5m models outperformed the other variant sin terms of mAP. YOLOv5n and YOLOv5m achieved the highest mAP, of 0.774 and 0.753, which showed that both models can detect objects more accurately compared to the other variants for our specific safety application trained on the SLS dataset.

**Figure 13:**
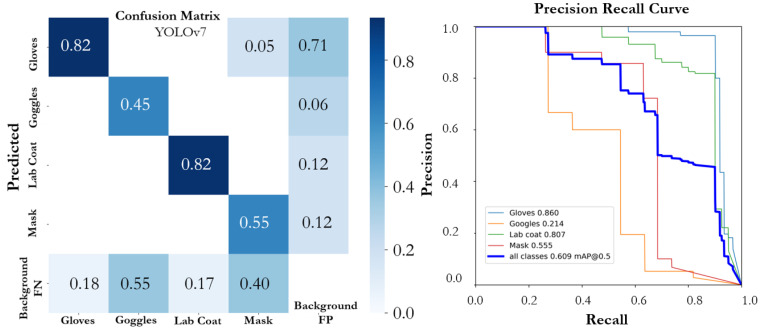
Confusion matrix and PR curve of the YOLOv7 model.

**Figure 14:**
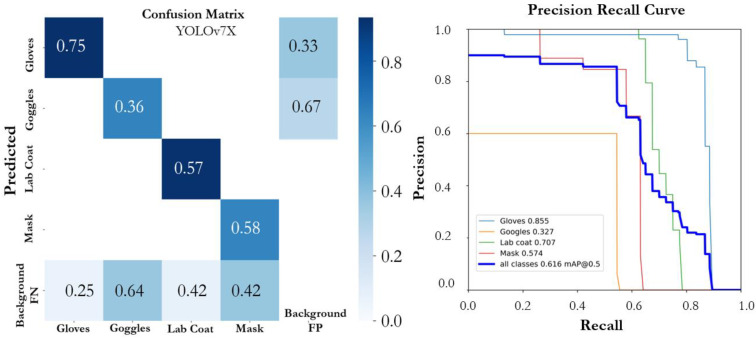
Confusion matrix and PR curve of the YOLOv7X model.

On top of that, YOLOv7 and YOLOv7X were evaluated for how well they performed on the proposed SLS dataset. The YOLOv7 model’s two variations did not demonstrate promising results. The YOLOv7X model outperformed the YOLOv7 model while achieving an mAP@0.5 of 0.616. The YOLOv7 model achieved precision, recall, and mAP@0.5 of 0.700, 0.654, and 0.609, respectively. The confusion matrices of both variants of YOLOv7 are shown in Figure 13 and Figure 14. YOLOv7’s accuracy was promising for the large-scale instances (lab coat and gloves classes); however, it was not as good for the small-scale instances (goggles and mask classes) due to the fact that the size of the class objects was small and there were fewer instances to train on. The YOLOv7 algorithm performed poorly on the proposed dataset when compared to the YOLOv5 algorithm on detecting small-scale instances, which is also in line with the literature [36]. Additionally, among the large-scale instances classes, gloves had the highest mAP@0.5, of 0.943, 0.952, 0.958, 0.954, 0.921, 0.860, and 0.855 corresponding to the model variants v5n, v5s, v5m, v5l, v5x, v7, and v7X, respectively. Among all the variants, YOLOv5m achieved the highest performance for all scale instances by achieving mAP@0.5 values of 0.958, 0.510, 0.872, and 0.672 for the gloves, goggles, lab coat, and mask classes, respectively. The two classes, mask and goggles, were not detected properly by all the variants of the YOLO models due to a small object size and lower number of instances of the classes in the data and a complex background of the training images.

**Figure 15:**
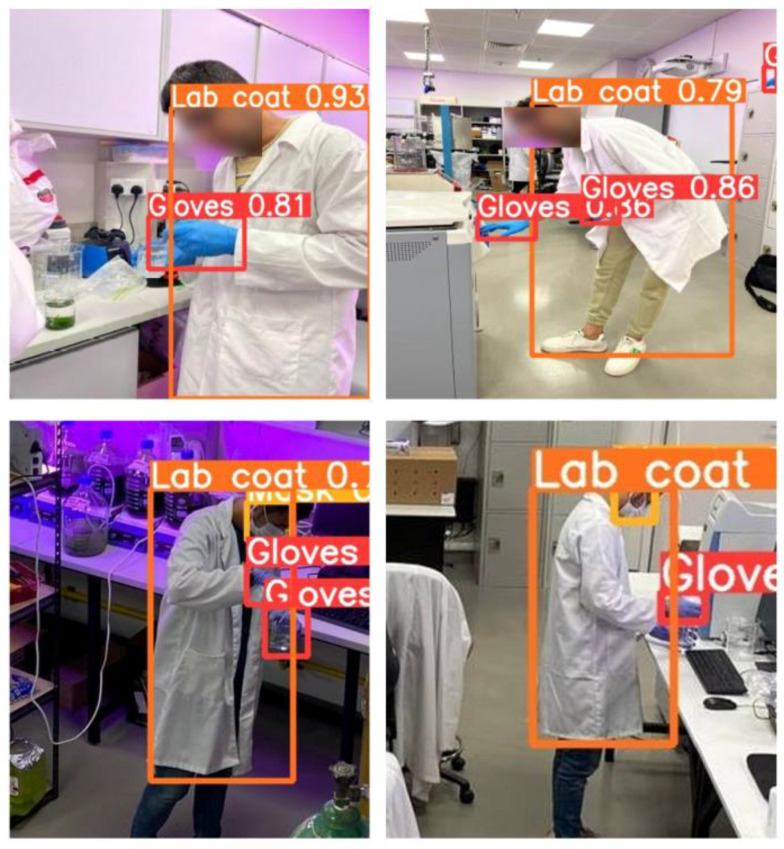
Testing results of the proposed system (YOLOv5m model).

The precision and recall curves of the models and the confusion metrics depict that the YOLOv5n model showed a high mAP@0.5 and true positives as compared to the other models. The real-time testing of the proposed system was performed on YOLOv5 models, and the results are shown in Figure 15. The images in the second row are the images acquired from the top camera installed in the lab; these show that the trained model can accurately predict the objects in the test dataset acquired from the surveillance camera. The acquired results showed that the system can be used in a lab environment for the compliance of student PPE. From the results, it was found that the YOLOv5n and YOLOv5m models outperformed the other variants of YOLOv5 in terms of performance and computational complexity. Both models have a smaller number of parameters and fast detection speed in comparison with their later variants. When comparing the YOLOv5 and YOLOv7 based on large-scale and small-scale instances, the YOLOv5 versions’ detection capabilities stand out. The YOLOv7’s overall accuracy suffered because it could not detect the small-scale items efficiently. This was due to the limited number of small-scale instances in the SLS dataset and complex background in the images. It was also found that increasing the network complexity had a significant effect on the performance and the speed of the models. It is also evident from Table 2 that YOLOv5l had less weight and number of parameters as compared to the other compared variants of YOLOv5, which helped in reducing the computational time and complexity of the models. Increasing the weights and number of parameters not only increased the computational time of the models but also led to degradation of the performance on this particular SLS dataset. The YOLOv5l and YOLOv5x models had the highest weight and number of parameters while achieving a lower detection accuracy and speed as compared to other YOLOv5 variants. Nevertheless, the experimental results showed that the YOLOv5 model guarantees high performance for the PPE detection of students in educational labs. Due to the paucity of relevant literature and scant efforts in the research community about PPE monitoring in educational labs, this work does not provide a comparative analysis of the proposed algorithms vs. state-of-the-art approaches.

## 4. Conclusions

In the proposed work, YOLOv5- and YOLOv7-based PPE compliance monitoring systems were implemented to enhance the safety of academic labs. Firstly, a dataset consisting of four various classes, i.e., lab coat, gloves, goggles, and mask, was created. Secondly, various variants of YOLOv5 and YOLOv7 were trained, and their performance was compared based on various evaluation metrics such as precision, recall, mAP, weights, and computational time of the algorithm. From the above discussion, it can be concluded that YOLOv5 and YOLOv7 models can be used for PPE detection of the student to provide lab instructors with more efficient and intelligent safety strategies. It can also be concluded that lightweight variants of YOLOv5 such as YOLOv5n and YOLOv5m can be utilized to build a robust and fast PPE detection system. The YOLOv5n and YOLOv5m achieved the highest mAP@0.5, of 0.774 and 0.753, respectively. The YOLOv5 variants were able to perform well for various scale instances as compared to the YOLOv7 model. Among all YOLOv5 models, the highest @mAP was achieved by the YOLOv5n and YOLOv5m. The YOLOv7 was not able to show a promising performance for small-scale objects due to the limited number of instances, the size of the objects, and the complex background of the acquired data. The performance of the models can be enhanced by providing more efficiently labeled data with a sufficient number of instances for all the classes. In conclusion, training a lightweight model with a sufficient amount of data is the best option for practical PPE detection systems. The proposed system will considerably reduce the occurrence of safety-related incidents and accidents in the labs by creating a safety culture. In addition, the proposed system will enhance the traditional lab safety training process by providing insights to the lab instructors about the safety protocols of the students, creating a feedback loop in which the information is best absorbed. In the future, we will improve the performance of YOLOv5 and YOLOv7 variants by using various optimization techniques and we will add more classes to the data to expand the applicability of the proposed system. The suggested system will also leverage methods such as the Internet of Things (IoT) and big data to make educational labs safer places to learn.

## Figures and Tables

**Table 1:** Comparison of the various YOLOv5 versions for PPE detection.

	Precision	Recall	mAP@0.5	mAP@0.5:0.95	Weights	No. of Parameters
YOLOv5n	0.795	0.787	0.774	0.485	3.9 MB	1.9 M
YOLOv5s	0.798	0.702	0.717	0.476	14.5 MB	7.2 M
YOLOv5m	0.837	0.776	0.753	0.481	42.3 MB	21.2 M
YOLOv5l	0.805	0.725	0.707	0.482	92.9 MB	46.5 M
YOLOv5x	0.794	0.688	0.725	0.488	173.2 MB	86.7 M
YOLOv7	0.700	0.654	0.609	0.366	74.8 MB	36.9 M
YOLOv7X	0.775	0.652	0.616	0.400	142.1 MB	71.3 M

**Table 2:** Individual class performance of the YOLOv5 detection models.

YOLOv5n					
Class	Instance Size	Precision	Recall	mAP@0.5	mAP@0.5:0.95
Gloves	L	0.918	0.918	0.943	0.610
Goggles	S	0.566	0.636	**0.565**	0.286
Lab Coat	L	0.937	0.925	**0.93**	0.602
Mask	S	0.761	0.67	0.659	0.440
**YOLOv5s**					
Gloves	L	0.942	0.902	0.952	0.638
Goggles	S	0.519	0.455	0.366	0.247
Lab Coat	L	0.968	0.775	0.907	0.620
Mask	S	0.763	0.677	0.645	0.400
**YOLOv5m**					
Gloves	L	0.934	0.929	**0.958**	0.629
Goggles	S	0.666	0.636	0.510	0.242
Lab Coat	L	0.93	0.825	**0.872**	0.622
Mask	S	0.819	0.713	0.672	0.431
**YOLOv5l**					
Gloves	L	0.957	0.951	0.954	0.668
Goggles	S	0.553	0.545	0.339	0.207
Lab Coat	L	0.966	0.719	0.915	0.644
Mask	S	0.743	0.684	0.621	0.408
**YOLOv5x**					
Gloves	L	0.963	0.902	0.921	0.617
Goggles	S	0.53	0.545	0.473	0.282
Lab Coat	L	0.906	0.675	0.866	0.612
Mask	S	0.776	0.632	0.641	0.439
**YOLOv7**					
Gloves	L	0.795	0.803	0.860	0.505
Goggles	S	0.483	0.364	0.214	0.079
Lab Coat	L	0.892	0.825	0.807	0.522
Mask	S	0.628	0.622	0.555	0.357
**YOLOv7X**					
Gloves	L	0.874	0.836	0.855	0.565
Goggles	S	0.599	0.544	0.327	0.162
Lab Coat	L	0.891	0.65	0.707	0.495
Mask	S	0.736	0.579	0.574	0.376

## Data Availability

Not applicable.
